# Heterogeneity in the Relationship between Disinfection By-Products in Drinking Water and Cancer: A Systematic Review

**DOI:** 10.3390/ijerph15050979

**Published:** 2018-05-14

**Authors:** Tarik Benmarhnia, Ianis Delpla, Lara Schwarz, Manuel J. Rodriguez, Patrick Levallois

**Affiliations:** 1 Department of Family Medicine and Public Health & Scripps Institution of Oceanography, University of California, San Diego, CA 92093, USA; lnschwar@ucsd.edu; 2 École Supérieure D’aménagement du Territoire et de Développement Régional (ESAD), Université Laval, 1624 Pavillon Savard, Québec, QC G1K-7P4, Canada; Ianis.Delpla@crad.ulaval.ca (I.D.); Manuel.Rodriguez@esad.ulaval.ca (M.J.R.); 3 Direction de la Santé Environnementale et de la Toxicologie, Institut National de Santé Publique du Québec, Québec, QC G1V 5B3, Canada; Patrick.Levallois@msp.ulaval.ca; 4 Axe Santé des Populations et Pratiques Optimales en Santé, Centre de Recherche du Centre Hospitalier Universitaire (CHU) de Québec, Québec, QC G1V 2M2, Canada

**Keywords:** THMs, cancer, effect measure modification, drinking water

## Abstract

The epidemiological evidence demonstrating the effect of disinfection by-products (DBPs) from drinking water on colon and rectal cancers is well documented. However, no systematic assessment has been conducted to assess the potential effect measure modification (EMM) in the relationship between DBPs and cancer. The objective of this paper is to conduct a systematic literature review to determine the extent to which EMM has been assessed in the relationship between DBPs in drinking water in past epidemiological studies. Selected articles (*n* = 19) were reviewed, and effect estimates and covariates that could have been used in an EMM assessment were gathered. Approximately half of the studies assess EMM (*n* = 10), but the majority of studies only estimate it relative to sex subgroups (*n* = 6 for bladder cancer and *n* = 2 both for rectal and colon cancers). Although EMM is rarely assessed, several variables that could have a potential modification effect are routinely collected in these studies, such as socioeconomic status or age. The role of environmental exposures through drinking water can play an important role and contribute to cancer disparities. We encourage a systematic use of subgroup analysis to understand which populations or territories are more vulnerable to the health impacts of DBPs.

## 1. Introduction

Disinfection is widely used for drinking water treatment to inactivate pathogens and to prevent waterborne diseases. This process can produce disinfection by-products (DBPs), which result from a chemical reaction between disinfectants such as chlorine and organic or inorganic matter in the water [1]. Among the various DBPs (>600) [2], trihalomethanes (THMs) are the most studied due to their relatively high prevalence and concentration in drinking water.

Numerous studies assess the association between exposure to chlorinated water and diverse health outcomes. Some of them investigate the influence of chlorinated water or THMs on reproductive health, such as small for gestational age [3,4], stillbirth, and low birth rate [3,5]. However, the majority of epidemiological studies investigate the impact of such water quality exposure on cancer outcomes. Epidemiological studies have shown that DBP exposure through drinking water is associated particularly with some types of cancer, namely, colon, rectal, and bladder [2,6,7,8], though contrasting results for colon and rectal cancers have been found [6]. Reviews revealing a consistent association between long-term exposure to THMs and risk of bladder cancer have been published in recent years [8,9,10]. Additionally, several epidemiological studies found other risk factors for colon, rectal, and bladder cancer, such as tobacco consumption [11,12,13,14,15], and dietary and genetic factors [13,16,17,18,19,20,21]. Other potential risk factors include urban residence [13,22] and alcohol consumption [13,16]. The duration of exposure is also associated with an increasing risk of bladder cancer [7].

In a pooled analysis published in 2004, it was observed that the effect of THMs on bladder cancer risk was more pronounced in men than women [23]. Several explanations were discussed, including residual confounding and biological plausibility such as sex differences in metabolizing DBPs. A proposed mechanism to explain this difference is the role of sex hormones in the modulation of enzymes that metabolize chlorination by-products (for chloroform and brominated THMs) into reactive metabolites. Other possible explanations are anatomic differences or variation in voiding frequency between men and women, which can influence the action of DBPs as hormone disruptors [23].

To our knowledge, besides the study described above, no systematic review has attempted to synthesize the literature that directly evaluates potential effect measure modifiers other than sex in the association between disinfection by-products in drinking water and cancer. Yet, several other potential effect measure modifiers such as socio-economic status could be particularly relevant to shaping policies that target vulnerable populations or territories and aim to reduce inequalities in cancer risks. This could help frame interventions to target specific subgroups and ensure exposure of DBPs, such as THMs and HAAs, are below harmful levels. Such assessments for populations that are unfairly treated in regard to their environmental exposures are relevant to the field of environmental justice, which has drawn recent attention with important concerns about other water contaminants such as with lead exposure in the Flint crisis [24]. In addition, these assessments can identify which populations and territories are more vulnerable to the harmful effects of exposure to THMs and inform interventions to reduce health disparities.

The literature in relation to environmental health inequalities emerged during these last two decades and distinguishes two types of environmental inequalities: (i) inequalities related to the level of exposure and (ii) inequalities related to the level of vulnerability (when the effect of an environmental exposure is modified according to different sub-groups strata of the population) [25,26,27,28].

Findings from studies investigating differential exposure have shown that deprived populations can be more exposed to contaminants in drinking water [5,24,29,30,31,32,33,34,35]. Among the few studies dedicated to the analysis of environmental inequalities associated with DBPs, all focus on inequalities related to the level of exposure, and show contrasting results. Briggs et al. [36] found a positive association between levels of THMs in drinking water and an index of multiple deprivation. Inversely, Delpla et al. [32] showed that municipalities with a lower material deprivation index have a lower risk of elevated THMs levels at their tap. Evans et al. [33] and Vrijheid et al. [35] found no significant relationships between individuals or community deprivation and THM concentrations in drinking water. The absence of an association could be linked to one or more of the following: geographical size of a study that could attenuate the associations when extending the studied area [36], lower participation among people of lower education, type of exposure studied, location of both the early-life and current residence of the person, and/or type of socio-economic indicator chosen [35].

This review focuses on differential vulnerability, which refers to the notion of “effect measure modification” in epidemiology. This concept of vulnerability can be defined as a “greater likelihood of an adverse outcome given a specific exposure, compared with the general population, including both host (individual) and environmental (contextual) factors” [37]. We will focus this review on socioeconomic status (SES) variables, but we will also consider potential vulnerability factors as sex/gender and health behaviors.

Differences in health opportunities and resources related to social class, race, and geographic area can lead to a lower health status for vulnerable groups [38]. For example, it has been shown in the last decade that that neighborhood SES can modify the effect of air pollutants [38,39] on mortality. Such evidence has been used to provide recommendations towards interventions aimed at specific low SES areas to reduce inequalities in mortality [40]. Understanding the effect measure modification (EMM) in the health impacts of DBPs will be useful in shaping policies to reduce cancer inequalities through proportionated interventions aimed at reducing exposure to DBPs. Yet, some drinking water distribution systems may span large geographic areas and serve large and diverse populations, so interventions on the distribution systems may be insufficient. In such a case, targeted local interventions in the infrastructure or through awareness campaigns may be relevant.

The overall objectives of this paper are to synthetize the literature studying the role of EMM in the relationship between DBPs in drinking water and cancer, and understand the extent to which it has been assessed in past epidemiological studies. If this epidemiological information is available in existing publications, this review will allow us to report the epidemiological evidence on the differential vulnerability of DBPs to cancer risk. If epidemiological information is lacking on this topic, the review will serve to highlight the knowledge gap and motivate future studies in this area. Two successive stages are performed: (i) An updated systematic review is conducted on studies measuring the association between DBPs in drinking water and colorectal and bladder cancers and (ii) The presence of EMM is evaluated according to socio-demographic characteristics and individual behaviors considered in the identified studies.

## 2. Materials and Methods

### 2.1. Search Strategy

We aim to identify all epidemiological studies investigating the effects of DBPs in drinking water on colorectal and bladder cancers published in English in scientific journals between January 1975 and August 2015. The strategy used to conduct this review, in accordance with the PRISMA guidelines [41], consisted of grouping keywords that represented (i) the exposure (namely DBPs in drinking water) and (ii) the health outcomes (namely, colon and rectal cancer (CRC) and bladder cancer). Keywords, titles, and abstracts were searched in PubMed and Elsevier Embase on the Ovid SP portal and Web of Science. There was no restriction on geographical location.

The keywords used for the literature search are as follows: (Disinfection by-products OR Disinfection-by-products OR water disinfection OR chlorination by-products OR water chlorination OR trichloromethane OR chloroform OR bromoform OR tribromomethane OR dichlorobromomethane OR dibromochloromethane OR THM OR Haloacetic acids) AND (colorectal cancer OR colorectal neoplasm OR colon cancer OR rectal cancer OR bladder cancer OR CRC).

Terms describing EMM were not included at this stage to avoid being too restrictive. Instead, we appraised the EMM assessment during the data extraction process (see below). We did not include keywords related to study design at this stage, but instead assessed this question while selecting studies (see below).

### 2.2. Selection of Studies

In the first stage, the first two authors of this paper read and screened the abstract of each returned article.

Papers meeting the following criteria were excluded from our review:

- Commentaries, editorials, review articles, or meta-analysis
- Studies not performed on human populations
- Studies not published in English
- Studies not including DBPs in drinking water or chlorinated water as the exposure
- Qualitative studies

When reviewers disagreed about whether a study should be excluded, the two met in person to discuss until an agreement was reached.

In a second stage, papers selected in the previous stage were fully screened and then excluded according to the following criteria:

- Studies not reporting a quantitative estimate between DBPs in drinking water or chlorinated waters and colorectal cancer or bladder cancer
- Studies not including colorectal cancer and/or bladder cancer as health outcomes
- Studies using an ecological design or only a spatial analysis

In addition, the reference section of studies identified was searched, and relevant references that were not initially identified were added.

### 2.3. Data Extraction

Selected articles were reviewed separately by the first two researchers, and each documented the first author, location, date of publication, sample size, study design (case-control, cohort study or case-cohort), exposure measurement, health outcomes assessed, effect size and CI (Confidence Interval), whether they included EMM assessment, and which subgroup was included in this assessment.

Finally, among studies that did not include an assessment of EMM, collected variables (i.e., those presented in the sample description used as confounders) that could potentially be used for an EMM assessment were reported. We included variables for which there is some evidence of EMM in other environmental determinants of population health and with documented mechanisms leading to a differential effect. We thus included the following variables: age [42,43,44,45], socio-economic factors [39,46], urbanization level [47,48], smoking status, and other health behaviors (alcohol consumption, diet, and physical activity) [49,50]. Sex was also included in the review. Socio-economic factors include SES variables such as education and occupation, as well as indexes that were used in the studies selected.

## 3. Results

### 3.1. Description of Studies Selected

**Figure 1:**
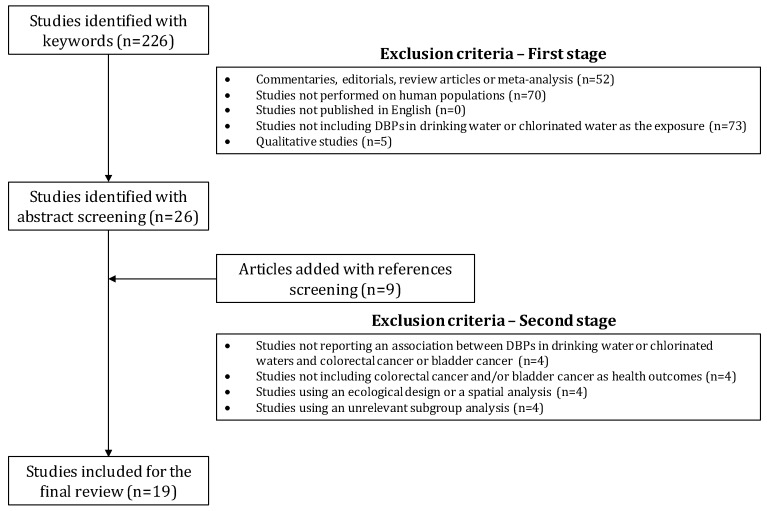
Flowchart of the selection of studies.

The abstracts of 226 articles were assessed, and 26 articles were retained for in-depth review after applying the first stage of exclusion criteria. Nine articles were added after screening the references of selected papers. Finally, 19 scientific articles were retained following the second stage exclusion criteria (Figure 1).

Table 1 summarizes the studies that provide a measure of the cancer risk associated with exposure to disinfection by-products through drinking water.

Eleven studies were conducted in North America, six in Europe, and two in Asia (Taiwan). The studies were published between 1981 and 2010. The majority of studies focus on bladder cancer (*n* = 14), followed by colon cancer (*n* = 4) and rectal cancer (*n* = 5). All are case-control studies, with the exception of Wilkins and Comsock, [51] and Koivusalo et al., [52] which are cohort studies. The majority of studies use values of THMs issued from field measurements (*n* = 10) or modeling (*n* = 3) to assess the exposure. The remaining ones use presence or absence of chlorinated water (*n* = 4) or the level of mutagenicity of waters (*n* = 2) as a marker of exposure to DBPs. THMs cut-offs varied between studies, because the exposure was calculated differently. Studies use long term (>30 years), fixed [53,54], or variable duration of exposure [55,56], although the majority use fixed levels (but different values) of DBPs [57,58,59,60,61,62]. The association between exposure and health issues is almost always assessed using logistic regression, with Odds Ratios (OR) or Risks Ratios (RR) being reported.

Odds Ratios in the studies selected are between 1.20 and 2.99 for bladder cancer, 0.90 and 1.66 for colon cancer, and 1.01 and 1.68 for rectal cancer (for OR calculated on the whole population under study). Fourteen studies found a significant relationship with cancer [52,53,55,57,58,59,60,62,63,64,65,66,67,68]. Five studies did not find a significant relationship between exposure to chlorinated water/DBPs and cancer, four of which studied bladder cancer [51,54,56,61] and one which studied colon cancer [69]. The majority of studies considered the exposure to THMs as a group of compounds without assessing the effects of the different compounds. Only the studies of Bove et al. [59,63] considered different species of THMs (chloroform, bromoform, bromodichloromethane, and chlorodibromomethane) in their analysis.

Approximately half of the studies assess EMM (*n* = 10), but the majority of them estimate it relative to different sex subgroups (total: *n* = 8, *n* = 6 for bladder cancer and *n* = 2 both for rectal and colon cancers). Generally, higher ORs and relationships that are more significant were noted for men when studying bladder and colon cancers. For example, in the study of Cantor et al. [64], an OR of 1.8 for men compared to an OR of 0.6 for women was found for bladder cancer. For rectal cancer, the results are contradictory, but the number of studies is too limited (*n* = 2) to draw any conclusion relative to sex. For women, only one study, which is also a cohort study [52], noted positive relationships for bladder and rectal cancer. Moreover, the study of Koivusalo et al. [52] is the only one that found higher risks for women than men (for the three different types of cancer). The duration of exposure was associated with an increase in bladder cancer risk for men only [7,64]. Fewer studies assessed the EMM relative to smoking status (*n* = 3). Finally, one study assessed EMM considering gene polymorphisms [62]. Generally, in all studies, the EMM assessment was conducted with stratified analyses. In the study of King et al. [57], an interaction term between exposure and sex was calculated.

### 3.2. Covariables Collected

Table 2 reports the variables collected that could be used for potential EMM assessment for each of the selected studies.

Although EMM is not assessed in the majority of the selected papers, some studies collect covariables that could be used in the future for an heterogeneity assessment. Information about age is commonly collected in selected studies (*n* = 16). Moreover, other individual data such as education and/or occupation status are collected in some of the studies (*n* = 11). Others report an SES index (*n* = 12). Information about the level of urbanization is also frequently collected (*n* = 9).

## 4. Discussion

The majority of studies assessing the relationship between THMs and cancer are case-control studies that focus on bladder cancer. These studies use exposure data issued from direct measurements or modelling. The majority of studies found an association between chronic exposure to THMs and bladder cancer. This review revealed that the majority of studies assessed EMM, but primarily for the effect of sex. Six studies show that the EMM of sex in the association between exposure to DBPs and bladder cancer may exist, with a higher effect for men than for women. However, only a very small number of studies have assessed this effect modification for other cancer sites such as colon and rectal cancer (*n* = 2 for both cancers), thus preventing any further conclusion about this parameter. Sex has been found as a more consistent EMM in bladder cancer, and several mechanisms have been proposed, as previously mentioned. More precisely, pharmacokinetic models in humans have shown that the activity of CYP2E1, which plays a role in chloroform metabolism, could be higher in men than in women [70,71]. The role of sex hormones in the modulation of enzymes that metabolizes DBPs has also been proposed. Brominated THMs are metabolized through a glutathione conjugation reaction and several studies have shown that glutathione transferases are regulated by thyroid and sex hormones [23].

The duration of exposure is also an important factor, as the outcomes are cancers at different sites with different latencies, and it is associated with an increase in bladder cancer risk for men [7,64]. Despite this, the exposure metrics (duration of exposure and means of exposure assessment) differ between studies selected. This could influence the results obtained in the different studies selected.

Furthermore, many epidemiological studies on DBPs collect other covariables in addition to sex, such as SES, age, urbanization level, and smoking status but without evaluating the EMM. This information could be used to conduct an EMM assessment in future studies and document the existence of vulnerability factors in the association between DBPs and risk of cancer (notably, bladder, colon, and rectal cancers). Of course, EMM assessments should be motivated and rely on a documented hypothesis. These assessments are particularly useful in targeting populations or territories that public policies should prioritize to reduce socio-demographic inequalities in cancer risks from water contaminants exposure. We thus strongly encourage further studies to assess the role of socio-demographic factors such as SES, age, or urbanization level as potential EMMs in the relationship between DBPs and cancer risk. It is also important to mention that various methods exist in the literature that assess EMM, such as the Breslow-Day test, the Wald χ^2^ test, and the regression-based test of interaction [72,73].

This review is subject to a number of limitations. We included studies that measured THMs exposure in a range of different approaches. THMs exposure measurement methods have drastically evolved in the last decades, but we decided to include older studies, as they can inform one of our aims to reveal possible EMM in the association between THMs exposure and cancer risk. We added 9 studies after the screening of references of existing papers. This may be due to some missing keywords (e.g., “treated drinking water”) despite the fact that we used similar keywords to other systematic reviews on this topic. Finally, some papers that have been recently published are not included in our review. For instance, a case–control study conducted in Spain and Italy by Villanueva et al. [74] assessed the impact of long-term exposure to THMs on colorectal cancer and found no association, considering the total population without including any EMM assessment.

The majority of the studies quantitatively include gender as a subgroup analysis, but very few studies focused on other potentially relevant EMMs such as SES. However, we observed that many studies included in the review collected standard details on covariates that could be, but often are not, used as stratifying variables. By doing so, we aim to highlight that EMM assessments are easily feasible in future studies using data that is already collected. We hope that this assessment will encourage future studies that will assess which populations are more vulnerable to the impacts of THMs exposure.

Studies on EMM are important to improve public health interventions and better estimate the potential benefits of an intervention (or its public health impact). For instance, within a large community/municipality that is supplied by a unique water treatment plant, inequalities in DBP exposure can be associated with the geographical location of deprived population groups within the municipality, living in neighborhoods or sectors with relatively high concentration of DBPs in drinking water (explained by variable residence time of water in the distribution network, pipe material, age and maintenance, or plumbing systems characteristics). In these cases, local interventions can reduce DBP exposure and could include two dimensions: (i) infrastructure: for instance, the renewal of distribution system pipes locally, including plumbing systems, the improving of the local hydraulic management of the system to reduce stagnation of water, or a better management of booster disinfection; and (ii) promoting awareness campaigns directed at deprived population concerning the exposure to DBPs through tap water and other domestic water uses as bath and showering (for example, the adequate use of domestic equipment to reduce DBPs, such as domestic water filtering, boiling, and refrigeration).

## 5. Conclusions

Inequalities in cancer incidence according to socio-demographic characteristics [75,76,77] or location (ex: rural areas compared to urban areas) have been highlighted in the literature. The role of environmental exposures such as drinking water contaminants can play an important role in such disparities. However, the documentation of EMM evidence is still lacking. We therefore strongly recommend greater use of subgroup analysis when possible, which will provide a greater understanding of which populations or territories are more vulnerable to the impacts of DBPs.

## Figures and Tables

**Table:** (**a**) Bladder cancer.

Studies	Location	Year of Publication	Sample Size	Study Design	Disinfection by-Products Measurement	Subgroups Included in the Analysis	Effect Size and CI
Wilkins and Comstock [51]	Maryland, USA	1981	81 cases and 30,699 controls	Cohort	Exposure to chlorinated drinking water	Sex	All: RR = 2.2 (0.71–9.39); Men: RR = 1.80 (0.80–4.75); Women: RR = 1.60 (0.54–6.32)
Zierler et al. [55]	Massachusetts, USA	1988	614 cases and 1074 controls	Case-control	Duration of exposure to chlorinated drinking water	n.d.	All: OR = 2.7 (1.7–4.3)
McGeehin et al. [53]	Colorado, USA	1993	327 bladder cancer and 261 other-cancer controls	Case-control	Questionnaire & data for TTHM from site visit of water utilities	Smoking Status	Non–Smokers: OR = 2.9 (1.2–7.4); Smokers: OR = 2.1 (1.1–3.8)
King and Marrett [67]	Ontario, Canada	1996	696 cases and 1545 controls	Case-control	Questionnaire about source of water. Water source and chlorination status were provided directly by treatment plant surveys. TTHMs modelling	n.d.	All: OR = 1.66 (1.11–2.51)
Freedman et al. [54]	Maryland, USA	1997	294 cases and 2326 controls	Case-control	Exposure to chlorinated drinking water	Sex, smoking habits	All: OR = 1.4 (0.7–2.9); Men: OR = 2.2 (0.8–5.1); Women: OR = 0.6 (0.2–2.2)
Koivusalo et al. [52]	Finland	1997	621 431	Cohort	Questionnaires. Information on water-pipe connections, past drinking water quality, and treatment practices by waterworks was obtained from administrative registers and municipal waterworks. The level of mutagenicity was estimated by modelling	Sex	All: RR = 1.12 (0.93–1.36); Men: RR = 1.03 (0.82–1.28); Women: RR = 1.48 (1.01–2.18)
Cantor et al. [64]	Iowa, USA	1998	1123 cases and 1983 controls	Case-control	TTHMs in tap water (measures + estimations)	Sex, smoking habits	All: OR = 1.3 (0.9–2.0); Men: OR = 1.8 (1.2–2.7); Women: OR = 0.6 (0.3–1.4)
Koivusalo et al. [56]	Finland	1998	732 cases and 914 controls	Case-control	Questionnaires. Information on water-pipe connections, past drinking water quality, and treatment practices by waterworks was obtained from administrative registers and municipal waterworks. The level of mutagenicity was estimated by modelling	Sex	Men: OR = 1.17 (0.87–1.57); Women: OR = 1.14 (0.71–1.82)
Chevrier et al. [58]	France	2004	281 cases and 272 controls	Case-control	TTHMs modelling	Sex	All: OR = 2.99 (1.1–8.5); Men: OR = 3.73 (1.2–11); Women: OR = 1.55 (0.1–32)
Bove et al. [59]	New York, USA	2007	182 cases and 385 controls	Case-control	TTHMs in tap water + water consumption	n.d.	THM: OR = 2.34 (1.01–3.66); CLF: OR = 2.55 (1.25–4.66); BRF: OR = 3.05 (1.51–5.69); BDCM: OR = 2.49 (1.19–4.48)
Chang et al. [60]	Taiwan	2007	403 cases and 403 controls	Case-control	TTHMs in tap water	n.d.	All: OR = 2.11 (1.43–3.11)
Michaud et al. [61]	Spain	2007	397 cases and 664 controls	Case-control	Questionnaire and records searches (including THM measurements)	n.d.	All: OR = 2.06 (0.83–5.08)
Villanueva et al. [68]	Spain	2007	1219 cases and 1271 controls	Case-control	Questionnaire and records searches (including THM measurements)	Sex	All: OR = 2.10 (1.09, 4.02); Men: OR = 2.53 (1.23, 5.20); Women: OR = 1.50 (0.26, 8.61)
Cantor et al. [62]	Spain	2010	680 cases and 714 controls	Case-control	TTHMs in tap water	Gene polymorphism	All: OR = 1.8 (0.9–3.5)

NB: n.d.: No data; OR: Odds Ratio; RR: Risk Ratio; CLF: Chloroform; BRF: Bromoform; BDCM: Bromodichloromethane; Q1: lowest THM concentrations quartile; Q4: highest THM concentrations quartile.

**Table:** (**b**) Colon and rectal cancers.

Studies	Location	Year of Publication	Sample Size	Study Design	Exposition Measurement	Site of Cancer	Subgroups Included in the Analysis	Effect Size and CI
Gottlieb and Carr [65]	Louisiana, USA	1982	546 cases and 534 controls	Case-control	Exposure to chlorinated drinking water	Rectal	n.d.	All: OR = 1.68 (1.17–2.42)
Koivusalo et al. [52]	Finland	1997	621 431	Cohort	Questionnaires. Information on water-pipe connections, past drinking water quality, and treatment practices by waterworks was obtained from administrative registers and municipal waterworks. The level of mutagenicity was estimated by modelling	Colon and rectal	Sex	Colon: All: RR = 0.90 (0.77–1.04); Men: RR = 0.83 (0.66–1.04); Women: RR = 0.95 (0.78–1.85). Rectal: All: RR = 1.04 (0.86–1.26); Men: RR = 0.85 (0.66–1.09); Women: RR = 1.38 (1.03–1.85).
Hildesheim et al. [66]	Iowa, USA	1998	560 colon cases, 537 rectal cases, and 1983 controls	Case-control	TTHMs in tap water	Colon and rectal	n.d.	Colon: OR = 1.06 (0.7–1.6); Rectal: OR = 1.66 (1.1–2.6)
King et al. [57]	Ontario, Canada	2000	767 colon cases, 661 rectal cases, and 1545 controls	Case-control	Questionnaire about source of water. Water source and chlorination status were provided directly by treatment plant surveys. TTHMs modelling	Colon and rectal	Sex	Colon: OR = 1.87 (1.15–3.05) for Men; OR = 0.92 (0.49–1.71) for Women. Rectal: OR = 0.98 (0.56–1.72) for Men; OR = 0.72 (0.34–1.53) for Women
Bove et al. [63]	New York State, USA	2007	128 cases and 253 controls	Case-control	TTHMs in tap water + water consumption	Rectal	n.d.	THM4: OR = 1.01 (0.98–1.03); CLF: OR = 1.00 (0.93–1.09); BRF: OR = 1.20 (1.05–1.35)
Kuo et al. [69]	Taiwan	2009	2195 cases and 2195 controls	Case-control	Questionnaire & data on TTHM levels in drinking water in study. Municipalities were collected from the Taiwan Environmental Protection Administration	Colon	n.d.	All: OR = 1.04 (0.89–1.21)

NB: n.d.: No data; OR: Odds Ratio; RR: Risk Ratio; CLF: Chloroform; BRF: Bromoform; BDCM: Bromodichloromethane.

**Table 2:** Co-variables collected in the selected studies (candidates for a potential effect measure modification assessment).

Study	Age	Sex	Socio-Economic Factors *	Urbanization Level	Smoking	Other Health Behaviors
Wilkins and Comstock 1981 [51]	X	X	X		X	
Gottlieb and Carr 1982 [65]	X	X				
Zierler et al. 1988 [55]				X	X	
McGeehin et al. 1993 [53]					X	
King and Marrett 1996 [67]	X	X	X		X	
Freedman et al. 1997 [54]	X		X	X		
Koivusalo et al. 1997 [52]	X	X	X	X	X	
Cantor et al. 1998 [64]	X	X	X		X	
Hildesheim et al. 1998 [66]				X		X
Koivusalo et al. 1998 [56]	X	X	X	X	X	
King et al. 2000 [57]	X	X	X			X
Chevrier et al. 2004 [58]	X		X	X	X	
Bove et al. 2007 [63]	X		X			X
Bove et al. 2007 [59]	X				X	X
Chang et al. 2007 [60]	X			X		
Michaud et al. 2007 [61]	X	X	X			
Villanueva et al. 2007 [68]	X	X	X	X	X	
Kuo et al. 2009 [69]	X	X		X		
Cantor et al. 2010 [62]	X	X	X		X	

* Include education, occupation status, or SES index.
